# Volume and Intensity of Locomotor Activity in International Men's Field Hockey Matches Over a 2-Year Period

**DOI:** 10.3389/fspor.2021.653364

**Published:** 2021-05-28

**Authors:** Carl A. James, Oliver R. Gibson, Aishwar Dhawan, Craig M. Stewart, Ashley G. B. Willmott

**Affiliations:** ^1^Sport Science Department, Institut Sukan Negara (National Sports Institute), Kuala Lumpur, Malaysia; ^2^Division of Sport, Health and Exercise Sciences, Center for Human Performance, Exercise and Rehabilitation (CHPER), Brunel University London, Uxbridge, United Kingdom; ^3^EDGE10 Group Ltd., London, United Kingdom; ^4^CS Performance, Clontarf Hockey Club, Dublin, Ireland; ^5^Cambridge Center for Sport and Exercise Sciences, Anglia Ruskin University, Cambridge, United Kingdom

**Keywords:** acceleration, speed, team sports, GPS, hockey, accelerometer

## Abstract

The locomotor demands of international men's field hockey matches were investigated across positions (DEF, MID, FWD) and playing quarters. Volume (i.e., total values) and intensity (i.e., relative to playing time) data were collected using 10-Hz GPS/100-Hz accelerometer units from the #11 world-ranked (WR) team, during 71 matches, against 24 opponents [WR 12 ± 11 (range, 1–60)]. Mean ± SD team total distance (TD) was 4,861 ± 871 m, with 25% (1,193 ± 329 m) “high-speed running” (>14.5 km h^−1^) and 8% (402 ± 144 m) “sprinting” (>19.0 km h^−1^). Reduced TD (range, −3 to 4%) and average speed (range, −3.4 to 4.7%) occurred through subsequent quarters, vs. Q1 (*p* < 0.05). A “large” negative relationship (*r* = −0.64) was found between playing duration and average speed. Positional differences (*p* < 0.05) were identified for all volume metrics including; playing duration (DEF, 45:50 ± 8:00 min; MID, 37:37 ± 7:12 min; FWD, 33:32 ± 6:22 min), TD (DEF, 5,223 ± 851 m; MID, 4,945 ± 827 m; FWD, 4,453 ± 741 m), sprinting distance (DEF, 315 ± 121 m; MID, 437 ± 144 m; FWD, 445 ± 129 m), and acceleration efforts (>2 m s^−2^; DEF, 48 ± 12; MID, 51 ± 11; FWD, 50 ± 14). Intensity variables similarly revealed positional differences (*p* < 0.05) but with a different pattern between positions; average speed (DEF, 115 ± 10 m min^−1^; MID, 132 ± 10 m min^−1^; FWD, 134 ± 15 m min^−1^), sprinting (DEF, 7 ± 3 m min^−1^; MID, 12 ± 4 m min^−1^; FWD, 14 ± 4 m min^−1^), and accelerations (DEF, 1.1 ± 0.3 n min^−1^; MID, 1.4 ± 0.2 n min^−1^; FWD, 1.5 ± 0.3 n min^−1^). Physical outputs reduced across playing quarters, despite unlimited substitutions, demonstrating the importance of optimizing physical preparation prior to international competition. Volume and intensity data highlight specific positional requirements, with forwards displaying shorter playing durations but greater high-intensity activities than defenders.

## Introduction

International hockey (sometimes referred to as “field hockey”) tournaments are characterized by a high “density” of matches, with the Olympic Games requiring players to play eight matches within 16 days, to win a medal. Thus, a comprehensive understanding of the physical demands of the game is necessary, to prepare players appropriately. Over the past two decades, hockey has undergone multiple rule changes. This includes, unlimited substitutions (1992), “fast” restarts (2009), the addition of a sixth outfield substitute (2013) and in 2015, changing the match format from two 35–min halves, to four 15–min quarters (International Hockey Federation, 2019). Emerging evidence indicates these changes have altered the locomotor demands, with an increased proportion of high-intensity activities (Ihsan et al., 2018; Morencos et al., 2018); however, the reporting of the locomotor demands of men's hockey since 2013 and 2015 remains limited.

The physical demands of hockey may be monitored using wearable miniature electro-mechanical devices, providing both global positioning system (GPS) and inertial measurement unit (IMU) data (Cummins et al., 2013). These devices provide distances traveled at different velocities (Willmott et al., 2018) and can quantify specific movements such as accelerations, decelerations, or changes of direction (James et al., 2021). Since the 2013/2015 rule changes, it has been suggested that players cover a similar total distance (~8,400 m) (Ihsan et al., 2018) within the modern game, compared with the historical 70-min format (~8,000–8,500 m) (Lythe and Kilding, 2011; Buglione et al., 2013). However, these data represent extrapolated data to the respective full match durations (60/70 min) and due to the shortened modern match duration, the absolute total distance (TD) covered by players at the 2016 Olympic Games was considerably lower [~5,600–6,500 m (McMahon and Kennnedy, 2019)]. Thus, whilst extrapolated data enables comparisons against other sports and/or hockey match formats, these data remain hypothetical and do not represent the actual activity experienced by players (Polglaze et al., 2018; Lombard et al., 2021). Nevertheless, there appears a greater proportion of high-speed running (>14/15 km h^−1^) within the modern game (Lombard et al., 2017; Ihsan et al., 2018). Coaches also recognize an increased importance of such high-speed running for successful performance, by implementing tactical rolling substitutions or “rotation” strategies, to maintain high physical outputs (Linke and Lames, 2017). Therefore, training methods that complement this trend appear popular, with international teams implementing repeated-sprint training to improve this specific aspect of performance (Brocherie et al., 2015; James and Girard, 2020). Increased high-speed running during matches is theoretically concurrent with additional high-intensity accelerations and decelerations. The monitoring of these actions is pertinent, as they are associated with the development of neuro-muscular fatigue and muscle damage in other team-sports, such as soccer (de Hoyo et al., 2016). Recent data indicates hockey players undertake ~100 “high-intensity” accelerations (Ihsan et al., 2018) and decelerations (Chesher et al., 2019) during international matches. During six World League matches, Ihsan et al. (2017) observed 1.2–1.5 accelerations and 1.3–1.7 decelerations per min^−1^, using a comparable threshold of 2 m s^−2^. However, the reporting of these movements in the modern game remains somewhat limited. Therefore, a comprehensive understanding of the locomotor demands of international hockey (i.e., running volume and intensity) at both a team and positional level is necessary, to facilitate effective training prescription, recovery strategies, and minimize injury risk (Bourdon et al., 2017).

Few studies have investigated both the volume and intensity of locomotor demands of international men's hockey since 2015. McMahon and Kennedy (2019) reported absolute (i.e., not extrapolated) TDs of 6,153 ± 990 m, 5,783 ± 810 m, and 5,451 ± 793 m for defenders (DEF), midfielders (MID), and forwards (FWD), respectively, across three tournaments (16 matches), which included the Olympic Games. The most high-speed distance (≥15 km h^−1^) was covered by MID (1,446 ± 264 m), followed by FWD (1,359 ± 296 m), and DEF (1,123 ± 373 m). When interpreted relative to typical playing durations, these data allude to positional differences in the locomotor demands of modern hockey. However, playing intensity data (i.e., relative to playing time) such as average speed [meters per minute (m min^−1^)], or the frequency of specific movements such as the total accelerations and decelerations, were not reported. Ihsan et al. (2018) collected data from a similar number of matches (*n* = 14), played by the Singapore national team [2018 world ranking (WR) #35–40]. Based upon data extrapolated to the full-match duration, the authors identified FWD as the most demanding position, followed by MID and finally DEF. However, the absolute distances covered by players were not reported nor average speed data without extrapolation. Therefore, different interpretations of the demands experienced by FWDs may arise from the dichotomous reporting of either relative (Ihsan et al., 2018) or absolute data (McMahon and Kennnedy, 2019). Average speed data without extrapolation had earlier been reported by Ihsan et al. (2017) from six international matches, indicating the range of team whole-match average values to be between 121 and 133 m min^−1^. However, the absolute volume (i.e., total values) and intensity (i.e., relative to time) of locomotor demands across different positions and the four quarters from a large number of matches, have yet to be reported in unison since the 2013 or 2015 rule changes. Consequently, relationships between total playing time and playing intensity, which may directly inform substitution strategies that seek to maintain physical outputs throughout matches, have yet to be identified. Moreover, reporting of the locomotor demands from a large sample of matches containing a variety of opponent and tournament standards is warranted. This reflects that the standard of competition elicits an independent effect on playing demands (Jennings et al., 2012a; McMahon and Kennnedy, 2019), which may bias data derived from smaller samples of matches.

Therefore, the aims of this study were to utilize data collected from a top-15 world-ranked team to; (i) comprehensively report GPS and IMU-derived volume and intensity of the locomotor demands across a large sample of international men's hockey matches, (ii) investigate differences in the whole-match locomotor demands between major positional groups (DEF, MID, FWD), (iii) investigate differences in the whole-team locomotor demands across playing quarters, and (iv) identify whether relationships existed between playing time and match intensity variables. We hypothesized that the locomotor demands would differ between positions, with FWD and MID completing more high-speed running, sprinting distance, and sprinting efforts, than DEF, yet locomotor outputs would reduce through the four quarters, and with increased playing time.

## Methods

### Experimental Approach to the Problem

A retrospective analysis was undertaken of 71 international matches, played across a two-year period (March 2018–November 2019). There were 24 different opponents (WR, 12 ± 11; range, 1–60). Each player participated in an average of 40 ± 20 matches (range, 9–66). Data were only derived from official test matches and tournaments including; Hockey World Cup, Asian Games, Asian Champions Trophy, and World Series Finals. Practice matches were excluded. Data are reported in terms of both volume (i.e., total values) and intensity [i.e., relative to playing time (per min)].

### Subjects

Twenty-seven male, international hockey players from the Malaysia national team (2018 WR #12, 2019 WR #11) participated in the study (nine DEF, seven MID, and 11 FWD; age 25 ± 4 years, stature 172 ± 5 cm, body mass 68 ± 6 kg, sum of seven skinfolds 45.3 ± 10.8 mm). Players typically undertook 10 training sessions per week, two strength training, and eight field sessions. Three field sessions contained a greater physical conditioning emphasis, one session was active recovery and the remaining four sessions a greater technical emphasis. The study had institutional ethical approval, and all analysis was conducted retrospectively on anonymous data, in accordance with the Declaration of Helsinki (2013).

### Procedures

Data were collected using a triaxial 10 Hz GPS/100 Hz accelerometer unit (G5 firmware v. 7.40, Catapult Sports, Australia) harnessed between the scapulae in a customized sports vest, with which players were familiarized from daily training. Wherever possible, players used the same device and vest. Equipment failures resulted in five players receiving replacement devices during the 2 years. For one tournament (six matches), players used different devices from the same manufacturer (Catapult S5, firmware v. 7.32). Devices were turned on and placed in the center of the pitch for 10 min prior to use. Where this was not possible (Hockey World Cup and World Series Finals), devices were worn throughout the warm-up (~40-min), which was followed by 4-min static, in the center of the pitch, for national anthems.

### Data Processing

Devices were downloaded using a *Catapult Sports* docking station and processed using *Openfield* software (version 2.3.3, build #52841). Match data were processed live, by the same individual. This involved excluding data associated with large breaks in play; between playing quarters, substitutions, sin-bins, penalty corners, video referrals, major injuries, and goal scoring (Ihsan et al., 2018). Therefore, data used for analysis pertains to situations where the game clock is running and may be considered “ball-in-play time.” The total playing time for each player was used to calculate their respective intensity data.

Running >14.5 km h^−1^ was classified as “high-speed running” and >19.0 km h^−1^ as “sprinting.” Accelerations and decelerations are reported from GPS data (Catapult Gen2) and classified as either “high-intensity” (HI, 2.0–3.5 m s^−2^) or “very high-intensity” (VHI, >3.5 m s^−2^) events. Total accelerations or decelerations were taken as the sum of HI and VHI events (i.e., all events >2 m s^−2^) as per current hockey literature (White and MacFarlane, 2015; Ihsan et al., 2018). Velocity and acceleration dwell times were 1.0 and 0.4 s, respectively.

Data from the IMU were used to calculate *Playerload* (Gómez-Carmona et al., 2020). Specific movements that were identified by the IMU (accelerations, decelerations, and left/right changes of direction) were similarly categorized as VHI if they exceeded 3.5 m s^−2^, and subsequently combined into a single metric of multi-directional load (total VHI movements) (James et al., 2021). This metric therefore encompassed all movements in the horizontal plane and was processed using inertial movement analysis (version 2) (Catapult Sports, 2019).

Measures of GPS quality, horizontal dilution of precision (HDOP, 0.75 ± 0.14) and satellite number (11.6 ± 0.8) (Malone et al., 2017) were considered excellent by the manufacturer guidelines. We discarded data not meeting the following inclusion criteria; minimum of nine outfield players (0 cases), no data recorded or values visually identified as a technological error (two cases), and minimum of seven satellites during match (one case). These inclusion criteria resulted in the removal of one player's GPS data for one match and two further player's IMU data for two different matches, resulting in a total of three match files being removed, leaving 1,106 whole-match files for analysis. Where errors were detected, the corresponding GPS or IMU data was retained if this was considered accurate (verified by three authors, independently). We did not apply a minimum playing time threshold because only nine playing-quarter records had a duration of <3 min per quarter (out of a total of 4,399 individual playing-quarter records), which was the minimum planned on-pitch “rotation” for any match across the 2-year period.

### Statistical Analyses

Data are presented as mean ± SD and were analyzed using SPSS (v.26, IBM, USA) with statistical significance as *p* < 0.05. All outcome variables were assessed for normality of distribution using histograms, boxplots, and measures of skewness and kurtosis, before analysis. Descriptive statistics, including the coefficient of variation (CV; [SD/mean]^*^100) and smallest worthwhile change (SWC; 0.2^*^SD) were calculated from whole-match averages (Hopkins, 2014). One-way ANOVA compared the whole-match volume and intensity data of DEF, MID, and FWD, with Bonferroni *post-hoc*. Where homogeneity of variance was not achieved, Welch's corrected *F* value was used to indicate statistical significance. Factorial repeated measures ANOVA were used to identify interaction effects (playing quarter ^*^ position), with Bonferroni *post-hoc*. Relationships between playing time and playing intensity were analyzed using Pearson product–moment correlations.

## Results

### Whole-Match Descriptive Data and Positional Differences

**Figure 1:**
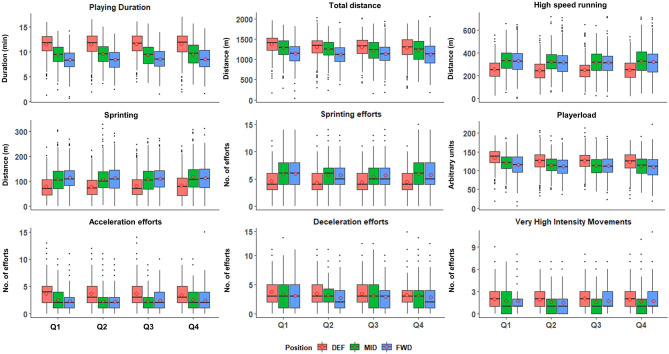
Volume of locomotor demands in international hockey across the four playing quarters and presented by playing positions. Data represent 4,399 individual playing-quarter match-records, derived from 71 international matches. DEF, defenders; MID, midfielders; FWD, forwards; Q1, first quarter; Q2, second quarter; Q3, third quarter; Q4, fourth quarter. Box edges represent 25 and 75% of the interquartile range (IQR). Horizontal lines within the box, median; circles within the box, mean; solid dots, outliers (>1.5 IQR); whiskers represent ±1.5 times *IQR. A.U., arbitrary units.

**Figure 2:**
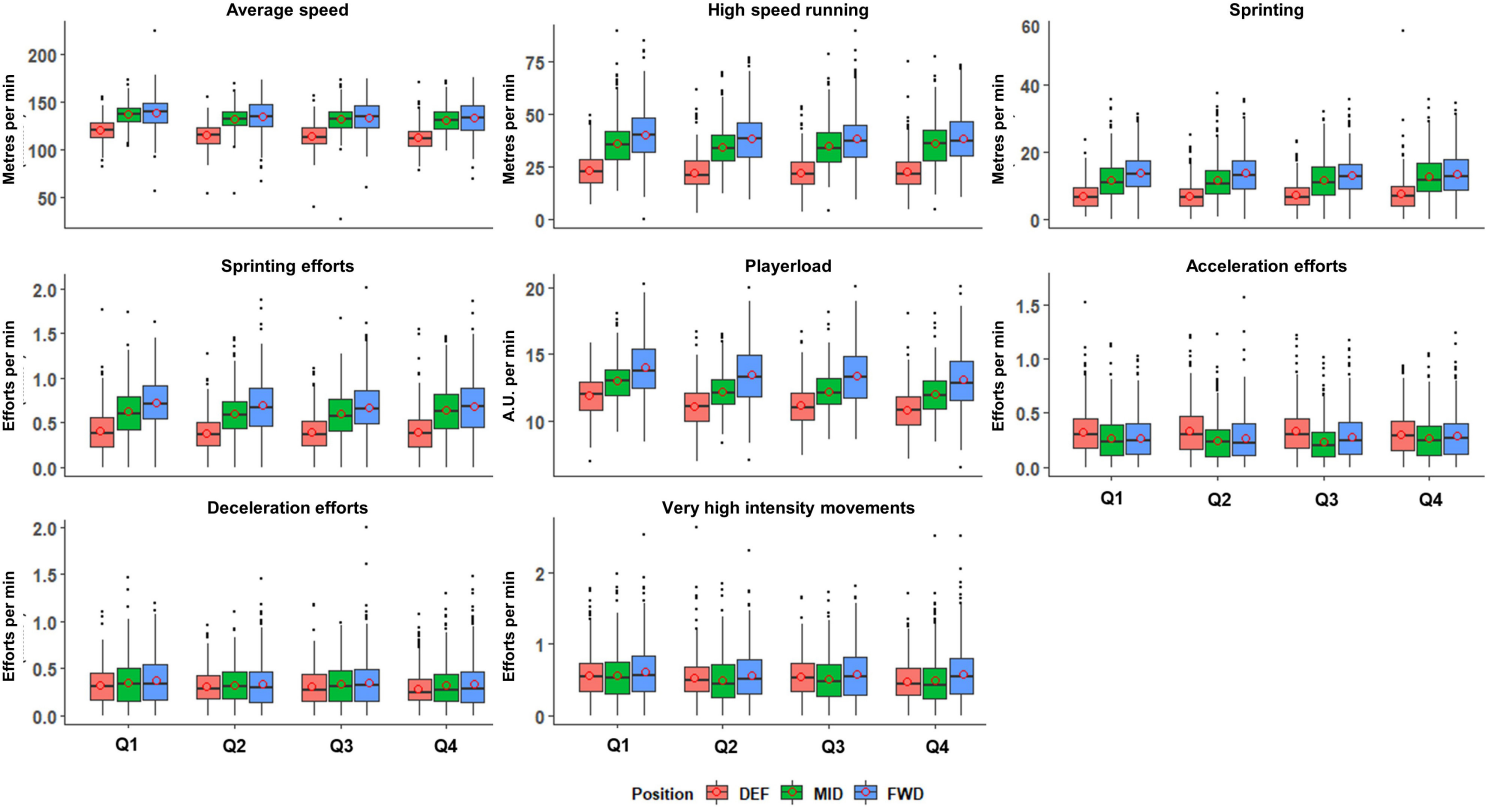
Intensity of locomotor demands in international hockey across the four playing quarters and presented by playing positions. Data represent 4,399 individual playing-quarter match records, derived from 71 international matches. DEF, defenders; MID, midfielders; FWD, forwards; Q1, first quarter; Q2, second quarter; Q3, third quarter; Q4, fourth quarter. Box edges represent 25 and 75% of the interquartile range (IQR). Horizontal lines within the box, median; circles within the box, mean; solid dots, outliers (>1.5 IQR); whiskers represent ±1.5 times *IQR. A.U., arbitrary units.

Descriptive reporting of whole-match volume and intensity data is displayed in Table 1. Figure 1 displays the distribution of volume data for all playing quarters, relative to playing position, of all players. Differences between positions in whole-match averages were observed for all variables (*p* < 0.05). Similarly, Figure 2 displays intensity data for all playing quarters, by playing position, and revealed positional differences within all variables (*p* < 0.05). For clarity, *post-hoc* comparisons between positions and effect sizes are provided in Table 2.

**Table 1:** Descriptive data for volume and intensity of whole match locomotor demands in international men's hockey.

	**Mean ± SD**	**CV%**	**Median**	**Range**	**IQR**	**SWC**
**Volume**						
Playing duration (min)	38:47 ± 08:49	23%	38:35	07:28–61:32	32:21–44:55	01:46
Total distance (m)	4,861 ± 867	18%	4,898	950–7,250	4,270–5,484	173
High-speed running (m)	1,191 ± 328	28%	1,190	228–2,331	970–1,399	66
Sprinting (m)	401 ± 144	36%	395	51–962	293–494	29
Sprinting efforts	21 ± 7	33%	21	2–49	16–26	1
Playerload (a.u.)	469 ± 84	18%	472	98–760	418–527	17
Acceleration efforts	50 ± 12	25%	49	11–98	42–57	2
Deceleration efforts	60 ± 14	24%	59	13–110	50–69	3
VHI movements	17 ± 8	44%	17	0–47	13–22	2
**Intensity**
Average speed (m min^−1^)	127 ± 15	12%	128	73–170	117–137	3
High-speed running (m min^−1^)	32 ± 11	33%	32	9–68	25–39	2
Sprinting (m min^−1^)	11 ± 5	41%	11	2–27	8–14	1
Sprinting efforts (n min^−1^)	0.6 ± 0.2	37%	0.6	0.1–1.3	0.4–0.7	0.04
Playerload with (a.u. min^−1^)	12.4 ± 1.9	15%	12.2	7.4–19.1	11.1–13.3	0.4
Acceleration efforts (n min^−1^)	1.3 ± 0.3	26%	1.3	0.4–2.6	1.1–1.5	0.1
Deceleration efforts (n min^−1^)	1.6 ± 0.4	25%	1.5	0.7–3.3	1.3–1.8	0.1
VHI movements (n min^−1^)	0.5 ± 0.2	41%	0.4	0.0–1.1	0.3–0.6	0.04

Data represent 1,106 individual player match-records, derived from 71 international matches. Acceleration and deceleration efforts identified using GPS data, when >2 m s^−2^. VHI movements is the sum of all movements in the horizontal plane (i.e., accelerations, decelerations, and left/right changes of direction), identified from the inertial sensor, that exceeded 3.5 m s^−2^. CV, coefficient of variation (CV; [SD/mean]*100); IQR, interquartile range; SWC, smallest worthwhile change.

**Table 2:** Whole-match positional data and Cohen's *d* effect sizes (ES; ±95% confidence interval) from 71 international matches.

	**Defenders**	**Midfielders**	**Forwards**	**DEF vs. MID**	**DEF vs. FWD**	**MID vs. FWD**
				**ES (95% CI)**	**ES (95% CI)**	**ES (95% CI)**
**Volume**						
Playing duration (min)	45:45 ± 08:05	37:37 ± 07:12^*^	33:32 ± 06:22^*^^#^	1.07 (0.91, 1.22)	1.69 (1.52, 1.86)	0.60 (0.46, 0.75)
Total distance (m)	5,223 ± 851	4,945 ± 827^*^	4,453 ± 741^*^^#^	0.33 (0.19, 0.48)	0.97 (0.82, 1.12)	0.63 (0.48, 0.78)
High-speed running (m)	998 ± 289	1,299 ± 298^*^	1,266 ± 310^*^	1.02 (0.87, 1.18)	0.89 (0.74, 1.04)	0.11 (−0.04, 0.25)
Sprinting (m)	315 ± 121	437 ± 144^*^	445 ± 129^*^	0.91 (0.76, 1.06)	1.03 (0.88, 1.19)	0.06 (−0.09, 0.20)
Sprinting efforts	18 ± 6	23 ± 7^*^	23 ± 7^*^	0.79 (0.64, 0.94)	0.82 (0.67, 0.97)	0.01 (−0.13, 0.15)
Playerload (a.u.)	507 ± 81	459 ± 76^*^	445 ± 83^*^^#^	0.60 (0.45, 0.75)	0.75 (0.60, 0.90)	0.19 (0.04, 0.33)
Acceleration efforts	48 ± 12	51 ± 11^*^	50 ± 14	0.22 (0.07, 0.36)	0.12 (−0.02, 0.26)	0.08 (−0.07, 0.22)
Deceleration efforts	61 ± 14	63 ± 15^*^	55 ± 13^*^^#^	0.20 (0.06, 0.35)	0.42 (0.27, 0.56)	0.61 (0.46, 0.76)
VHI movements	20 ± 8	16 ± 7^*^	16 ± 7^*^	0.56 (0.41, 0.71)	0.60 (0.46, 0.75)	0.04 (−0.11, 0.18)
**Intensity**						
Average speed (m min^−1^)	115 ± 10	132 ± 10^*^	134 ± 15^*^	1.73 (1.57, 1.9)	1.50 (1.34, 1.66)	0.14 (0, 0.29)
High-speed running (m min^−1^)	22 ± 7	35 ± 8^*^	39 ± 10^*^^#^	1.78 (1.61, 1.95)	1.93 (1.76, 2.11)	0.40 (0.25, 0.54)
Sprinting (m min^−1^)	7 ± 3	12 ± 4^*^	14 ± 4^*^^#^	1.41 (1.25, 1.57)	1.84 (1.67, 2.01)	0.44 (0.29, 0.59)
Sprinting efforts (n min^−1^)	0.4 ± 0.1	0.6 ± 0.2^*^	0.7 ± 0.2^*^^#^	1.39 (1.23, 1.55)	1.72 (1.55, 1.89)	0.44 (0.29, 0.59)
Playerload per min (a.u. min^−1^)	11.2 ± 1.3	12.4 ± 1.3^*^	13.4 ± 2.1^*^^#^	0.86 (0.71, 1.01)	1.26 (1.10, 1.42)	0.61 (0.46, 0.76)
Acceleration efforts (n min^−1^)	1.1 ± 0.3	1.4 ± 0.2^*^	1.5 ± 0.3^*^^#^	1.09 (0.93, 1.24)	1.32 (1.16, 1.48)	0.45 (0.31, 0.60)
Deceleration efforts (n min^−1^)	1.3 ± 0.3	1.7 ± 0.3^*^	1.7 ± 0.4^*^	1.15 (0.99, 1.30)	0.93 (0.78, 1.08)	0.09 (−0.06, 0.23)
VHI movements (n min^−1^)	0.5 ± 0.2	0.4 ± 0.2	0.5 ± 0.2^#^	0.13 (−0.01, 0.27)	0.14 (0, 0.29)	0.26 (0.11, 0.4)

* *Different from defenders*.

# *Different from midfielders (forwards only)*.

*DEF, defenders; MID, midfielders; FWD, forwards. Acceleration and deceleration efforts identified using GPS data, when >2 m s^−2^. VHI movements is the sum of all movements in the horizontal plane (i.e., accelerations, decelerations, and left/right changes of direction), identified from the inertial sensor, that exceeded 3.5 m s^−2^*.

### Between Playing Quarters

**Figure 3:**
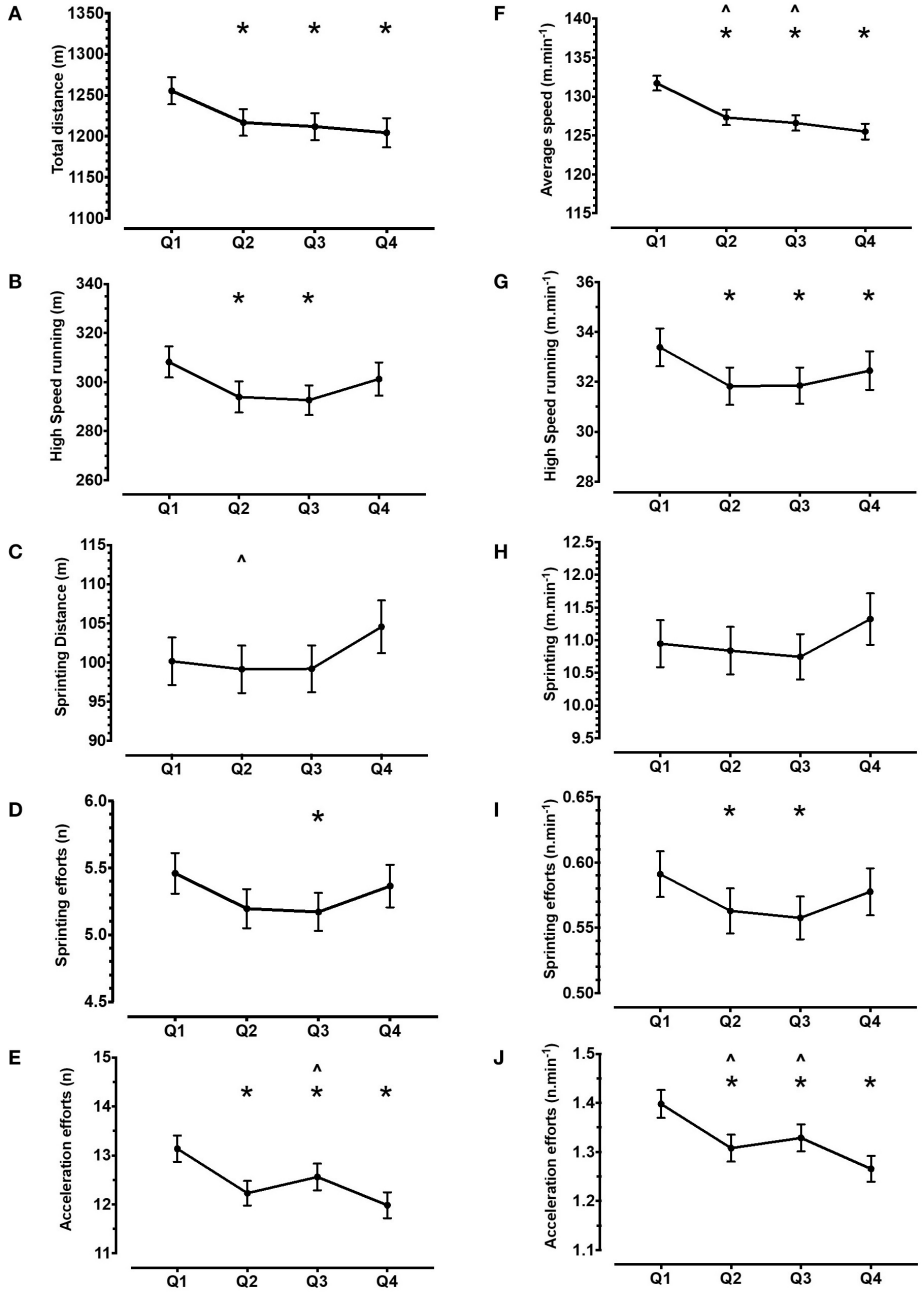
Team average of the volume (left panel, plots **A–E**) and intensity (right panel, plots **F–J**) of locomotor demands across playing quarters. Error bars represent 95% confidence interval. Data represent 4,399 individual playing-quarter match records, derived from 71 international matches. Asterisk represents difference vs. Q1, Circumflex accent symbol represents difference vs. Q4 (*p* < 0.05).

Analysis of volume data revealed statistical differences between quarters for TD, high-speed running, sprinting distance, sprinting efforts, *Playerload*, accelerations, decelerations, and VHI movements (all *p* < 0.05). *Post-hoc* analysis is displayed in Figure 3. Similarly, analysis of intensity data revealed differences between playing quarters for average speed, high-speed running per minute, sprinting efforts per minute, *Playerload* per minute, acceleration efforts per minute, deceleration efforts per minute, and VHI movements per minute (all *p* < 0.05, Figure 3). There was no difference between quarters for playing duration or sprinting distance per minute (*p* > 0.05).

### Relationships Between Playing Duration and Match Intensity

**Figure 4:**
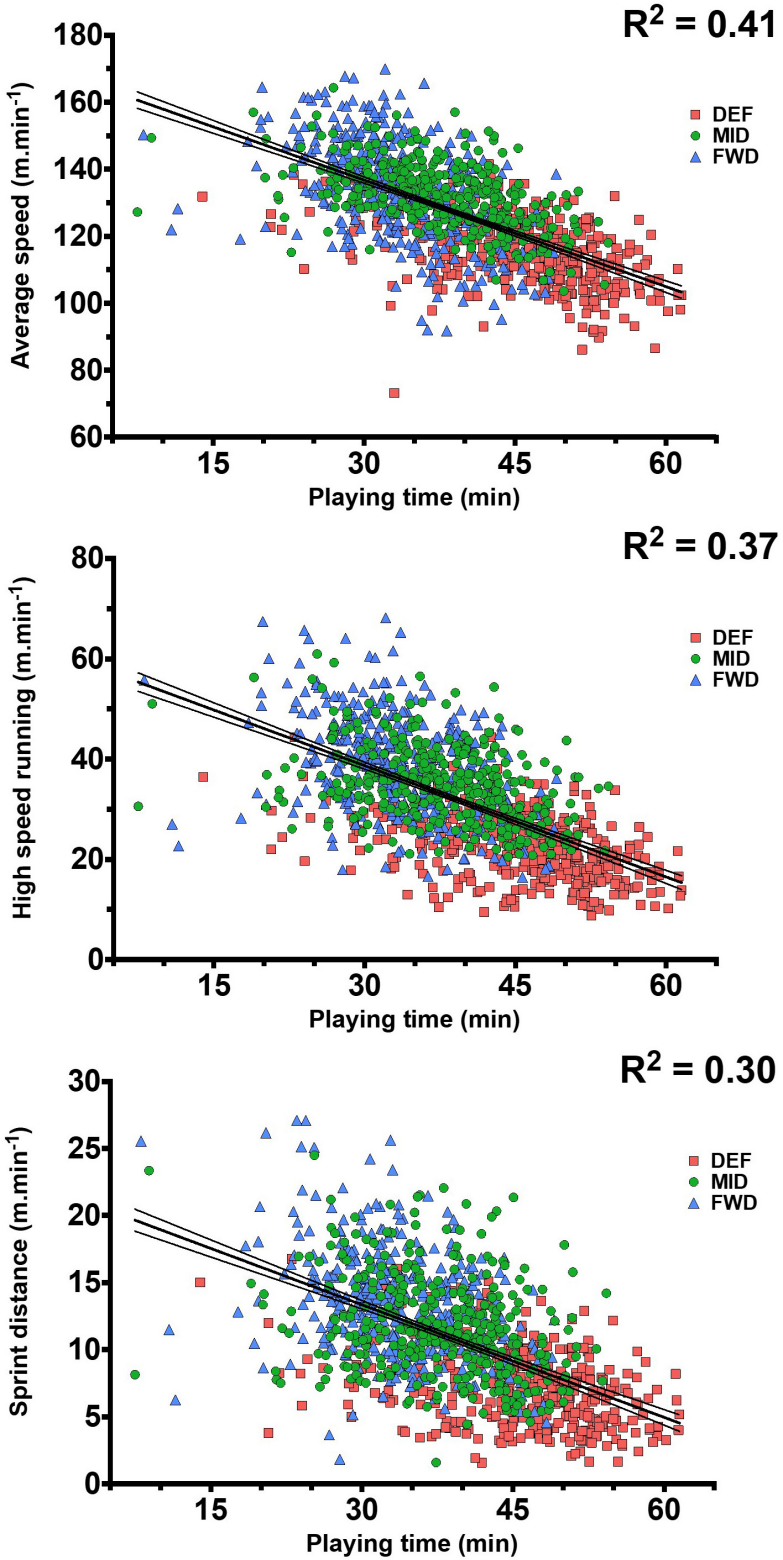
Relationships between playing time and average speed **(Top)**, high-speed running per minute **(Middle)**, and sprinting distance per minute **(Bottom)**. Data derived from 71 international men's hockey matches (*n* = 1,106 samples). Dashed lines represent 95% confidence interval around trendline.

The strongest relationship (±95% confidence interval) with playing duration was observed for average speed (*r* = −0.64 [±0.04], Figure 4). There were also relationships (all *p* < 0.001) with high-speed running per minute (*r* = −0.61 [±0.04]), *Playerload* per min (*r* = −0.61 [±0.04]), sprinting (*r* = −0.55 [±0.04]), sprinting efforts (*r* = −0.53 [±0.04]), acceleration efforts per minute (*r* = −0.51 [±0.04]), and deceleration efforts per minute (*r* = −0.49 [±0.04]).

## Discussion

This study utilized data from a top-15 world-ranked team to comprehensively report the volume and intensity of locomotor demands in international men's hockey matches by playing position and quarters. Our findings, from 1,106 individual player records and 71 matches, indicate the first playing quarter (Q1) has the highest demands. We observed reductions in both TD (range, 3–4%) and average speed (range, 3.4–4.7%) through subsequent quarters, compared with Q1. Players performed 50 ± 12 accelerations and 60 ± 14 decelerations per game, equivalent to performing a high-intensity movement once or twice per minute (Table 1). Differences in playing durations and demands between positions existed, with DEF spending more time on the pitch (+18% vs. MID, +27% vs. FWD) accruing larger total distances (+5% vs. MID, +15% vs. FWD), but generally playing at a lower intensity than MID and FWD (m min^−1^; −15% vs. MID, −17% vs. FWD). Finally, we observed strong negative relationships between total playing time and intensity metrics, reinforcing the need for effective physical training and recovery practices, as well as structured substitution policies, to maintain physical outputs throughout a match.

### Whole-Match Demands

At the team level, players covered 4,861 ± 871 m per game, with 25% (1,193 ± 329 m) considered high-speed running (>14.5 km h^−1^) and 8% (402 ± 144 m) sprinting (>19.0 km h^−1^). These total and high-speed distances are comparable with the limited data pertaining to international men's hockey since the 2013 and 2015 rule changes (Ihsan et al., 2017; McMahon and Kennnedy, 2019). However, the TD that we report is considerably lower than previous extrapolated data (FWD, 8,922 ± 818 m; MID, 8,613 ± 406 m; DEF, 7,631 ± 753 m) (Ihsan et al., 2018), despite the same game format and apparent similarity of data processing (i.e., removal of time-outs and penalty corners). This supports previous suggestions that extrapolation does not effectively characterize the true demands experienced by players (Polglaze et al., 2018; Lombard et al., 2021). Our data represent the absolute mean and range of values that may occur during international matches. As we did not apply inclusion criteria based upon a minimum playing time, the larger values within the ranges we report are highly pertinent for training benchmarks. These data may be considered “worst-case scenarios” when players experience additional demands, for example, when a teammate is injured or sent to the sin-bin (Table 1).

The observations that 28% and 8% of TD involves running >14.5 and >19.0 km h^−1^, respectively, reaffirm pre-2015 observations that hockey remains an intermittent high-intensity sport, whereby periods of low-intensity running are interspersed with high-intensity efforts (Lythe and Kilding, 2011). The sprinting distance (8%) exceeds the 6% previously reported from the New Zealand men's team in 2011 (Lythe and Kilding, 2011). While modest, this supports a trend towardz a greater proportion of high-speed running and sprinting in the modern game (Ihsan et al., 2018; McGuinness et al., 2019b; McMahon and Kennedy, 2019). Furthermore, the team's average speed (127 ± 15 m min^−1^), exceeds historical data from other international teams, e.g., 116 m min^−1^ (no SD reported) (New Zealand, 2011 WR #7) (Lythe and Kilding, 2011) and 124 m min^−1^ (95% CI, 120–128) (Scotland, 2015 WR #26) (White and MacFarlane, 2015). It is comparable with that of 12 players from the Singapore national team in 2015 (121–133 m min^−1^) (Ihsan et al., 2017). Nevertheless, the team average speed data are below that of the then world number 1 Australia team, during the two-half match format (131 ± 11 m min^−1^) (Polglaze et al., 2015), which may reflect a difference in physical outputs of the higher ranked sides vs. the current team (Gabbett, 2013).

The current data support previous suggestions that the standard of competition may influence playing demands (McMahon and Kennnedy, 2019), with the team's average speed higher than the Spanish domestic league (105–123 m min^−1^) (Romero-Moraleda et al., 2020). However, intensity data must be interpreted with caution, as a falsely high playing intensity may be derived if only time “in-play” data are analyzed, as this negates rest periods associated with events such as; goal scoring, substitutions, or between quarters/half-time (White and MacFarlane, 2013). Therefore, our “ball-in-play” data may represent a higher intensity than players experienced during the match. However, it should be noted that failing to remove data associated with breaks in-play, may misrepresent the typical intensities experienced when the ball is in play. Finally, our approach enables comparisons to be made between matches and across sports, indicating hockey players maintain higher average speeds than in other team sports such as soccer, rugby union, and rugby league, but lower than Australian rules football (Aughey, 2011; Cummins et al., 2013).

### Positional Differences

Our data reveal FWD to play for the shortest duration (33 ± 6 min), accumulating lower TD than both MID (37 ± 7 min) and DEF (45 ± 8 min) but display higher intensities (Figure 1). These differences in playing durations are consistent with observations from men's (Jennings et al., 2012b) and women's international hockey pre-2015 (McGuinness et al., 2019b), as well as post-2015 women's hockey (McGuinness et al., 2019a). While the SD of playing time for DEF (±8 min) is larger than MID (±7 min) and FWD (±6 min), all positions are comparable when expressed as a CV (i.e., relative to average playing time; 18–19%; Table 1). This indicates similar variability of playing time across positions, despite the team's planned rotation strategy sometimes involving fewer DEF substitutions and thus potential for longer playing times during situations such as sin-bins or injuries.

Our findings that FWD achieved the lowest (absolute) TD and *Playerload* somewhat contrasts with previous conclusions from extrapolated data, that FWD have the highest “running demands” (Ihsan et al., 2018). This reinforces the importance of reporting and interpreting both the constituent parts of locomotor demands, i.e., the volume and intensity of work completed by players, for effective load management. The lower average speed of DEF has previously been linked to a higher playing duration (Polglaze et al., 2015). Indeed, we identified a strong negative relationship between total playing time and average speed (*r*^2^ = 0.41, Figure 4) across the 71 matches. This relationship may be considered “large” (Hopkins et al., 2009) and highlights the importance of a structured substitution policy, to balance playing time across the team and facilitate higher intensities across playing quarters (Linke and Lames, 2017).

We observed differences in the volume of high-intensity activities between positions, with DEF completing less high-speed running, sprinting meters, and sprinting efforts, than both MID and FWD, who did not differ (Figure 1). McMahon and Kennnedy (2019) reported comparable high-speed running demands (classified as ≥15 km h^−1^), with this representing ~18% of TD for DEF, ~25% for MID, and ~25% for FWD. This compares with our high-speed running (≥14.5 km h^−1^) percentages of 19% (DEF), 26% (MID), and 28% (FWD). However, when expressed by playing time, FWD completed more of these high-intensity activities than MID, who in-turn completed more than DEF (Figure 2). While a similar pattern was found for accelerations per minute (FWD, 1.5 ± 0.3; MID, 1.4 ± 0.2; DEF, 1.1 ± 0.3, Figure 2), the total VHI movements detected by the IMU did not reveal the same pattern, with DEF completing more VHI movements than MID (Figures 1, 2). This indicates a position-specific demand on DEF, independent of high-speed or locomotor running distances, as has been reported in soccer DEF (Dalen et al., 2016). In hockey, these demands likely include DEF performing shorter movements and/or changes of direction to eliminate an opponent's available space, making tackles or intercepting passes, thereby accruing more VHI movements.

### Comparisons Between Playing Quarters

We observed reductions in the TD (range, 2.9–4.0%) during Q2, Q3, and Q4, compared with Q1 (Figure 3). Similarly, average speed reduced across playing quarters, compared with Q1 (range, 3.4–4.7%), with the lowest values during Q4. Reduced running outputs have been observed in men's hockey within both older (Lythe and Kilding, 2011; Jennings et al., 2012a) and modern match formats (Ihsan et al., 2018; Morencos et al., 2018). Interestingly, this is despite unlimited available substitutions. Unlike TD, high-speed metrics such as high-speed running distance, sprinting distance, and sprinting efforts revealed “U-shaped” responses (Figure 3), with the highest values observed during Q1 and Q4. This alludes to situational factors, such as score-line or tactical strategy, influencing high-speed running demands, in the absence of a linear reduction, which would be consistent with physiological fatigue. Indeed, the nature of the reduction in running outputs is not well-understood. We found the number of high-speed events per minute did not change across playing quarters. This indicates the higher volumes during Q1 and Q4 are interspersed with longer periods of lower intensity running, which is supported by the consistent decline in average speed. Ihsan et al. (2018) suggested players may pace throughout a game by altering low-intensity movements, thereby preserving higher speed running efforts. In contrast, Morencos et al. (2018) found Spanish domestic league players cover similar TD, but less high-speed distance and fewer sprinting efforts as the game progressed. Therefore, the pattern of running outputs across matches appears to vary between teams, likely due to factors including physical conditioning, team ranking, opponent ranking, and tactical strategy. Future research is therefore warranted into the effect of situational factors such as score-line and environmental conditions on these locomotor demands across a match.

### Limitations

Our conclusions are drawn from only one team and may be susceptible to bias from the playing style and physiological characteristics of these players. Nevertheless, the inclusion of many opponents, standards of tournaments, and locations within our dataset somewhat mitigates these potential sources of bias. Moreover, the team had two different coaches within this period, who implemented different tactical approaches. Teams who implement different substitution strategies (i.e., not one DEF, two MID, and three FWD replacements) may observe different demands between positions or, less equitable playing time between individual players. Our investigation is focused upon whole-match or playing-quarter averages. Thus, periods of higher intensity activity may be observed within playing quarters. Future analysis should therefore consider within-playing-quarter activity patterns by analyzing player rotation durations (McGuinness et al., 2021) and/or peak passages of play (Delves et al., 2019; McGuinness et al., 2020). Finally, our categorization into three positions neglects discrete differences within positions, such as the role of wing-backs or holding midfielders. However, as players often play multiple positions as part of the rotation policy, this confounds further positional differentiation.

## Practical Applications

These data demonstrate the need for practitioners and coaches managing player's physical loads to consider both the playing volume and intensity, in order to understand the true physical demands players experience during matches. These data can be used to design training programs containing suitable running volumes to prepare players for tournaments with a high match “density,” e.g., up to eight match distances (~4,818 m) across an Olympic 13-day tournament (~38,000 m). The additional high-speed running completed by MID and FWD, compared with DEF, would exceed what may be considered the SWC in high-speed running for the whole team (66 m). Furthermore, when players repeat these loads eight times in 13 days, the accumulated difference would exceed the average of one game for all positions (Table 1). Therefore, position-specific conditioning would appear relevant when preparing for tournaments with multiple matches, such as the Olympics. Match intensity that is derived from whole-match averages, may provide training benchmarks, but risks under preparing players for the intensity experienced during individual quarters or shorter, intense passages of play (Delves et al., 2019; McGuinness et al., 2020). Thus, consideration should be given to identifying and replicating demanding passages of play within training, for suitable match preparation. As Q1 appears the most demanding quarter, there appears a need for optimizing pre-match priming strategies, nutrition, and warm-ups (McGowan et al., 2015). Furthermore, as some metrics display a U-shaped response, within-match strategies are also important. Finally, for effective player preparation, we highlight that replicating external match intensities we report, may not be optimal for stimulating specific, desirable physiological adaptations (Impellizzeri et al., 2019). Accordingly, the complimentary monitoring of internal training load is recommended, to understand the physiological stimulus that seeks to meet the demands of competition we describe.

## Data Availability Statement

The data analyzed in this study was subject to the following licenses/restrictions: direct any queries to the lead author. Requests to access these datasets should be directed to Carl A. James, carlalexanderjames@gmail.com.

## Ethics Statement

The studies involving human participants were reviewed and approved by Institut Sukan Negara. The patients/participants provided their written informed consent to participate in this study.

## Author Contributions

CJ, OG, CS, AD, and AW conceived and designed the study, analyzed and interpreted the data, drafted and revised the manuscript, and approved the final version of the manuscript. CJ collected the data. All authors contributed to the article and approved the submitted version.

## Conflict of Interest

AD was employed by the company Edge 10 Ltd. The remaining authors declare that the research was conducted in the absence of any commercial or financial relationships that could be construed as a potential conflict of interest.
